# Quadrature Squeezing and Geometric-Phase Oscillations in Nano-Optics

**DOI:** 10.3390/nano10071391

**Published:** 2020-07-17

**Authors:** Jeong Ryeol Choi

**Affiliations:** Department of Electrophysics, College of Convergence and Integrated Science, Kyonggi University, Yeongtong-gu, Suwon, Gyeonggi-do 16227, Korea; choiardor@hanmail.net

**Keywords:** geometric phase, squeezed state, one-photon process, nano-optics

## Abstract

The geometric phase, as well as the familiar dynamical phase, occurs in the evolution of a squeezed state in nano-optics as an extra phase. The outcome of the geometric phase in that state is somewhat intricate: its time behavior exhibits a combination of a linear increase and periodic oscillations. We focus in this work on the periodic oscillations of the geometric phase, which are novel and interesting. We confirm that such oscillations are due purely to the effects of squeezing in the quantum states, whereas the oscillation disappears when we remove the squeezing. As the degree of squeezing increases in *q*-quadrature, the amplitude of the geometric-phase oscillation becomes large. This implies that we can adjust the strength of such an oscillation by tuning the squeezing parameters. We also investigate geometric-phase oscillations for the case of a more general optical phenomenon where the squeezed state undergoes one-photon processes. It is shown that the geometric phase in this case exhibits additional intricate oscillations with small amplitudes, besides the principal oscillation. Such a sub-oscillation exhibits a beating-like behavior in time. The effects of geometric-phase oscillations are crucial in a wide range of wave interferences which are accompanied by rich physical phenomena such as Aharonov–Bohm oscillations, conductance fluctuations, antilocalizations, and nondissipative current flows.

## 1. Introduction

Geometric phases are ubiquitous. They have been observed in a wide variety of optical/mechanical systems [1,2,3,4,5,6]. The geometric phases can be used as an excellent tool for analyzing the characteristics of optical phenomena because they reflect the geometries of quantum states with enormous generality. Moreover, these novel phase properties can be applied to many different branches of physical sciences. In particular, the geometric phases in nano-optics are important because they are, in most cases, closely connected to next generation technologies [2]. The most familiar subject along these lines which the geometric phase plays a major role in, is fault-tolerant quantum computation of which operations are based on geometric phase gates [2]. Besides that, other disciplines in nano-optics, which adopt the geometric phases as a key factor, are geometric phase lenses [3], holograms [4], tunable beam-shift technology [5], virtual/augmented realities [6], etc.

More than two decades ago, nano-optics emerged as a new paradigm in nanoscience including condensed matter science, which provides a promising perspective for leading the continuous development of optics-based science and industry. An efficient coupling of optical light with nano-materials is now feasible through its tight localization in nano-dimensions which are, especially, smaller than the diffraction limit of visible light. While there is still an ambiguity regarding some mechanisms in nano-optics, such as Fano-like interference effects [7] and nano-step measuring [8], the development of exceptional abilities in controlling light waves with geometric phases is necessary in the fields of quantum sensing, information processing, and metrology.

In this work, we focus our attention on investigating the time evolution of the geometric phase in a squeezed state of light waves in nano-optics [9,10]. As is well known, the squeezed state is the generalization of the coherent state which is the most fundamental classical-like quantum state. Various experimental methods for generating squeezed states of light have been devised so far. They are based on currently accessible optical means and instruments, such as modified microring resonators [11], optical parametric amplifiers [12], and semiconductor diode lasers [13]. Squeezed states enable us to reduce variance in one quadrature below that of a coherent state via the increase of the variance in the other quadrature. This leads to enhancement of the SNR (signal to noise ratio) in optical communications. Moreover, such reduced uncertainty in a quadrature is crucial in science and technology even beyond optical communications [14]. Squeezing the state of a light within the domain comparable to subwavelength is required in diverse nano-optic manipulations with optical probes [15], quantum dots [16], CMOS [17], etc. Hence, it is highly desirable to clarify the characteristics of light waves, including the geometric phase in the generalized quantum states, the so-called squeezed states.

We will show that the geometric phase for the squeezed light in nano-systems described by the harmonic oscillator exhibits a novel distinct oscillation in time. Such oscillations of the geometric phase may significantly affect the pattern of interference phenomena [18] which are intrinsic in fundamental optical devices, such as interferometry [19], polarimeter [20], and microscopy [21]. We will also extend our theory to more complicated optical phenomena where the squeezed state undergoes one-photon processes [22]. It will be shown that one-photon mechanisms induce additional geometric-phase oscillations with small amplitudes. The time behavior of such oscillations, as well as the common oscillations, will be analyzed in detail from the fundamental level.

## 2. Results and Discussion

### 2.1. Description of the Squeezed State

A harmonic oscillator description [15,23] and/or a coupled oscillator description [24] for an optical light and light–matter interaction provides a fundamental building block for quantum devices in the field of quantum engineering associated with quantum information science. We consider a simple wave with the angular frequency ω in nano-optics, which propagates in a medium of which electric permittivity is ϵ. The vector potential in this case can be represented as

(1) $$A\left(r,t\right)=\sum_{l}u_{l}\left(r\right)q_{l}\left(t\right).$$

For convenience, let us drop the subscript *l* by considering only a particular polarization mode. While the polarization mode of u(r) is determined by the geometry of the space, q(t) follows the equation of motion $d^{2}q\left(t\right)/dt^{2}+\omega^{2}q\left(t\right)=0$. We can represent the classical solution in this case as

(2) $$q_{\mathrm{cl}}=C\cos\left(\omega t+\theta \right),$$

where *C* is the amplitude and θ a phase.

As we know, the optical phenomena are described by wave functions in quantum mechanics. As a preliminary step before the main development of the geometric phase, we will describe the squeezed state, including the associated wave function. The structure of the wave functions determines the geometric phase, while the wave function in the squeezed state is somewhat different from other states. We start from the Hamiltonian of the light wave:

(3) $$\hat{H}=\hbar \omega \left({\hat{a}}^{†}\hat{a}+1/2\right),$$

where the annihilation operator is given by $\hat{a}=\sqrt{\epsilon \omega /\left(2\hbar \right)}\hat{q}+i\hat{p}/\sqrt{2\epsilon \omega \hbar }$ with $\hat{p}=-i\hbar \partial /\partial q$. Before we see the squeezed state, let us first consider the coherent state which is more fundamental than the squeezed state. The coherent state |α〉 obeys the eigenvalue equation of the annihilation operator, such that $\hat{a}|\alpha \rangle =\alpha |\alpha \rangle .$ From this equation, we have the eigenvalue α in the form

(4) $$\alpha \left(t\right)=\alpha \left(0\right)e^{-i\omega t},$$

where $\alpha \left(0\right)=\alpha_{0}e^{-i\theta }$ with $\alpha_{0}=\sqrt{\epsilon \omega /\left(2\hbar \right)}C$.

On the other hand, the squeezed state is the eigenstate of an operator $\hat{b}$ which is defined as $\hat{b}=\mu \hat{a}+\nu {\hat{a}}^{†},$ where μ and ν are complex parameters that obey ${\left|\mu \right|}^{2}-{\left|\nu \right|}^{2}=1.$ For convenience, we chose the complex parameters such that [10]

(5) $$\mu =e^{-i\lambda }\cosh|\xi |\;\;\;\;\;\;\;\;\;\nu =e^{-i(\lambda -\varphi )}\sinh\left|\xi \right|,$$

where $\xi =\xi_{0}e^{i\varphi }$, and λ, $\xi_{0}$, and φ are real constants.

We can express the eigenvalue equation for the operator $\hat{b}$ as $\hat{b}|\beta \rangle =\beta |\beta \rangle ,$ where |β〉 is the eigenstate which corresponds to the squeezed state. The eigenstate 〈q|β〉 in the configuration space is the corresponding wave function. We have provided the exact formula of 〈q|β〉 with its detailed derivation in Methods section (which is the last section, i.e., Section 4).

Using the expectation values given in Section 4, the fluctuations of the canonical variables in the squeezed state can be easily identified to be

(6) $${\left(\Delta q\right)}_{\beta }=\sqrt{\frac{\hbar }{2\epsilon \omega }}S_{-}\;\;\;\;\;\;\;\;\;\;\;\;\;\;{\left(\Delta p\right)}_{\beta }=\sqrt{\frac{\epsilon \omega \hbar }{2}}S_{+},$$

where $S_{\pm }={\left(2|\nu \right|}^{2}+1\pm \mu \nu \pm \mu^{*}\nu^{*}{)}^{1/2}$. Thus, the fluctuations in the squeezed state are determined by μ and ν. Equation (6) will be used in analyzing the geometric phase in the next subsection.

### 2.2. Geometric Phase and Its Oscillation

If the overall phase of the wave function in a system does not return to its initial phase after a cyclic evolution of the wave, an additional phase as well as the familiar dynamical phase appears. Such an additional phase accumulation in the wave function is the geometric phase. To understand the mechanism associated with light waves with a deeper insight, we need to study geometric phases.

If we denote the phase of a quantum wave for a general system as γ(t), the wave function can be written in the form

(7) $$\psi \left(q,t\right)=\phi \left(q,t\right)e^{i\gamma (t)}.$$

Generally speaking, γ(t) is composed of a dynamical part and a geometric one. The geometric phase at an arbitrary time *t* for a system described by a general Hamiltonian, including a harmonic-oscillator-type one, can be defined as (see, e.g., references [25,26])

(8) $$\gamma_{G}\left(t\right)=\int_{0}^{t}\langle \phi \left(t^{\prime }\right)|i\frac{\partial }{\partial t^{\prime }}|\phi \left(t^{\prime }\right)\rangle dt^{\prime }+\gamma_{0},$$

where $\gamma_{0}$ is the geometric phase at initial time. Because $\gamma_{G}\left(t\right)$ is determined by the time derivative of |ϕ〉 (see Equation (8)), a geometric phase exists only when |ϕ〉 is a function of time. For example, for the standard description of the harmonic oscillator in the Fock-state, |ϕ〉 is not a function of time in configuration space such that

(9) $$\langle q|\phi_{n}\rangle =\sqrt[{4}]{\delta /\pi }{\left(\sqrt{2^{n}n!}\right)}^{-1}H_{n}\left(\sqrt{\delta }q\right)\exp\left[-\delta q^{2}/2\right],$$

where $H_{n}$ are Hermite polynomials and δ=mω/ℏ. In this case, the geometric phase does not exist [27]. On one hand, there is another opinion regarding the definition of the geometric phase in the Fock state and its corresponding outcome (see, e.g., Section 6.3.2 in [28]).

The concept mentioned above may also be applied to the description of light waves, because the Hamiltonian of a light wave is represented in terms of a harmonic-oscillator Hamiltonian, as shown in, for example, [29] (see also Equation (3)). The geometric phase of a light wave in the squeezed state is given by replacing |ϕ(t)〉 in Equation (8) with |β(t)〉, such that

(10) $$\gamma_{G,\beta }\left(t\right)=\int_{0}^{t}\langle \beta \left(t^{\prime }\right)|i\frac{\partial }{\partial t^{\prime }}|\beta \left(t^{\prime }\right)\rangle dt^{\prime }+\gamma_{0}.$$

On one hand, the geometric phase can also be defined in a slightly different manner, such that it is the phase of geometric origin accumulated during only a period of the eigenstate evolution along a closed circuit [30]. We will use the first definition of the geometric phase (Equation (10)) throughout this work due to its obvious advantage compared to the latter, in analyzing the time evolution of phase angles of geometric origin.

If we use the identity operator $\int_{-\infty }^{\infty }dq|q\rangle \langle q|=I$, Equation (10) can be rewritten as

(11) $$\gamma_{G,\beta }\left(t\right)=i\int_{0}^{t}dt^{\prime }\int_{-\infty }^{\infty }dq\langle \beta \left(t^{\prime }\right)|q\rangle \frac{\partial }{\partial t^{\prime }}\langle q|\beta \left(t^{\prime }\right)\rangle +\gamma_{0}.$$

Then, by utilizing Equation (31) with Equations (32) and (33) in Methods section (Section 4), the integration with respect to *q* in the above equation results in

(12) $$\int_{-\infty }^{\infty }dq\left[\langle \beta |q\rangle \frac{\partial \langle q|\beta \rangle }{\partial t}\right]=-i\omega \left(\frac{\left(\mu \alpha -\nu \alpha^{*}\right)\left(\alpha +\alpha^{*}\right)}{\mu -\nu }-\frac{\alpha^{2}}{\left(\mu -\nu \right)\left(\mu^{*}-\nu^{*}\right)}\right).$$

Using this relation and the formula of α given in Equation (4), we confirm that Equation (11) becomes

(13) $$\begin{array}{cc} \gamma_{G,\beta }\left(t\right)= & \frac{\omega \alpha_{0}^{2}}{\left(\mu -\nu \right)\left(\mu^{*}-\nu^{*}\right)}\int_{0}^{t}[1-2\left(\mu \nu^{*}e^{-i(\omega t^{\prime }+\theta )}+\mu^{*}\nu e^{i(\omega t^{\prime }+\theta )}\right)\cos\left(\omega t^{\prime }+\theta \right)\\ & +{4|\nu |}^{2}\cos^{2}\left(\omega t^{\prime }+\theta \right)]dt^{\prime }+\gamma_{0}. \end{array}$$

Now, by carrying out the integration with respect to *t* in the above equation, we easily have

(14) $$\begin{array}{cc} \gamma_{G,\beta }\left(t\right)= & \frac{\alpha_{0}^{2}}{\left(\mu -\nu \right)\left(\mu^{*}-\nu^{*}\right)}\{\omega t-(\mu \nu^{*}+\mu^{*}\nu -{2|\nu |}^{2})\\ & \times \left\{2\omega t-\sin\left(2\theta \right)+\sin\left[2\left(\omega t+\theta \right)\right]\right\}/2-i\left(\mu^{*}\nu -\mu \nu^{*}\right)\\ & \times \sin\left(\omega t\right)\sin\left(\omega t+2\theta \right)\}+\gamma_{0}. \end{array}$$

This is the geometric phase in the squeezed state. The evaluation of the geometric phase with *p*-representation also gives the same result (see Section 4 for the detailed evaluation).

**Figure 1:**
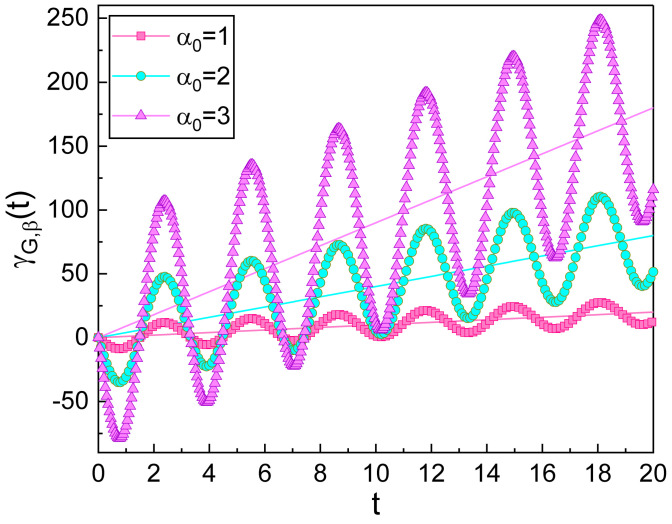
Temporal evolution of the geometric phase in the squeezed state for several values of $\alpha_{0}$. We have used $\xi_{0}=1.5$, λ=0, ω=1, φ=θ=0, and $\gamma_{0}=0$. The reference lines (solid straight lines) are geometric phases drawn without quadrature squeezing for each, i.e., depicted with the choice of $\xi_{0}=0$. By comparing all curves, we confirm that the amplitude of the geometric-phase oscillation gradually grows according to the increase of $\alpha_{0}$ under a quadrature squeezing.

From Figure 1, we see a novel feature that the geometric phase in the squeezed state oscillates over time. Let us see why this oscillation occurs. If μ→1 and ν→0, the squeezing effects disappear and the wave becomes the coherent state. In this case, Equation (14) reduces to [30,31,32]

(15) $$\gamma_{G,\alpha }\left(t\right)=\omega \alpha_{0}^{2}t+\gamma_{0}.$$

We see from the above equation that the geometric phase in the coherent state does not oscillate. Hence, it is obvious that the geometric-phase oscillation in Figure 1 originated entirely from the squeezing of the quantum state of the light wave. Squeezed states indeed exhibit rich novel physical properties that accompany the reduction of a quadrature uncertainty, which cannot be explained by classical mechanics or even from the semiclassical level [14].

In the coherent-state limit, although the oscillation of the geometric phase disappears, the phase increases linearly over time as can be seen from Equation (15), where the gradient of the increase is proportional to the square of the displacement $\alpha_{0}$. Therefore, the time behavior of the geometric phase in the squeezed state is the combination of the linear increase and the periodic oscillation as can be seen from Figure 1. In addition, the effects of $\alpha_{0}$ on the geometric phase can also be confirmed from Figure 1. As $\alpha_{0}$ becomes large, not only does the slope of the envelope of the oscillation grow, but interestingly, the amplitude of such an oscillation is also augmented. If we use another definition of the geometric phase which is the geometrical origin of phase accumulation during only a period *T*, then the result of the geometric phase resembles that in the coherent state, which does not undergo oscillation. Hence, it is impossible to confirm the oscillation of the phase, mentioned above, in that case.

**Figure 2:**
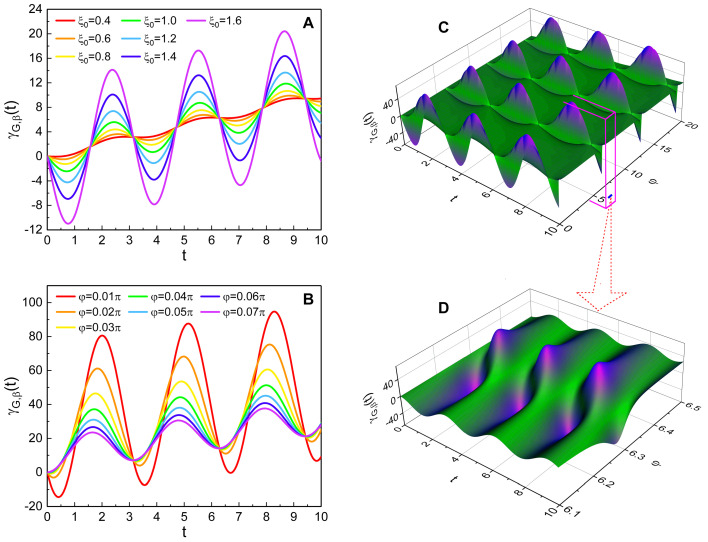
Temporal evolution of the geometric phase for several values of $\xi_{0}$ (**A**) and φ (**B**). (**C**) is 3D plot of the geometric phase versus φ and *t*. (**D**) is an enlarged view of a part of (**C**), which is within 6.1≤φ≤6.5. We used ($\alpha_{0}$, φ) = (1, 0) for (**A**), and ($\alpha_{0}$, $\xi_{0}$) = (1.5, 2) for (**B**–**D**). All other values are common and given by λ=0, ω=1, θ=0, and $\gamma_{0}=0$. From this figure, we can confirm the effects of squeezing parameters [$\xi_{0}$ (**A**) and φ (**B**–**D**)] on the geometric-phase oscillation.

The squeezing parameters μ and ν are represented in terms of $\xi_{0}$, φ, and λ (see Equation (5)). Figure 2 shows that the amplitude of the geometric-phase oscillation varies depending on the values of $\xi_{0}$ and φ. Hence, it is possible to control the oscillation amplitude of the geometric phase by adjusting $\xi_{0}$ and φ when $\alpha_{0}$ is fixed. However, λ does not affect the geometric phase because it appears in both μ and ν in the same manner. From Figure 2A, we can confirm that the oscillation amplitude of $\gamma_{G,\beta }\left(t\right)$ increases as $\xi_{0}$ becomes large. On the other hand, Figure 2B shows that the amplitude of such an oscillation decreases as the phase φ increases within the given range of φ in the graphic. The pattern of the geometric phase oscillation is significantly different depending on the value of φ.

Because the degree of the quadrature squeezing is determined by $\xi_{0}$ and φ, the geometric-phase oscillation in the squeezed state is closely related to the strength of the squeezing. Table 1 shows that the strength of *q*-squeezing increases as $\xi_{0}$ becomes large and/or φ becomes small. By comparing this fact with the outcomes of Figure 2A,B, we can conclude that the geometric-phase oscillation is amplified as the degree of *q*-squeezing increases.

More general behavior of the geometric phase depending on φ can be seen from Figure 2C,D. It shows that the geometric phase highly oscillates near φ=2πk where k=0,1,2,⋯; the amplitude of such an oscillation rapidly collapses as φ departs from those values. In fact, the decrease of the amplitude of the geometric-phase oscillation in Figure 2B along the augmentation of φ can be interpreted based on this. The departure of the value of φ from zero(=2π×0) is responsible for such an amplitude drop in that case.

**Figure 3:**
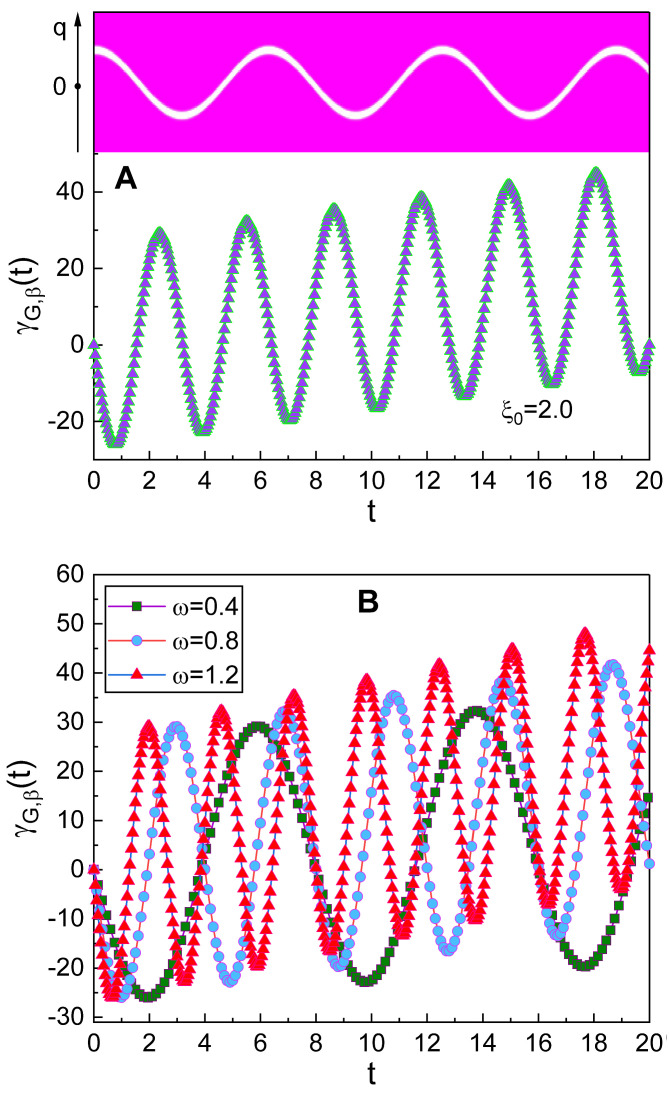
Comparison of the period of the geometric-phase oscillation with other fundamental periods related to the wave. (**A**): The geometric-phase oscillation (lower part) and the oscillation of the corresponding probability density ${\left|\langle q|\beta \rangle \right|}^{2}$ (upper part) in *q* space. We have used $\xi_{0}=2.0$, ϵ=1, and ℏ=1, while the values of all other data used in the plot are the same as those in Figure 2A. (**B**): Temporal evolution of the geometric phase in the squeezed state for several values of ω. We have used $\alpha_{0}=1$, $\xi_{0}=2$, λ=0, φ=θ=0, and $\gamma_{0}=0$.

The geometric-phase oscillation over time is compared with the corresponding probability-density oscillation in Figure 3A. The period of the geometric-phase oscillation is roughly one half of that of the oscillation in the probability density. Hence, if we regard that the frequency of the probability-density oscillation is ω, the frequency of the geometric-phase oscillation is approximately twice of ω. From Figure 3B, we can more clearly see the relation between the frequency of the geometric-phase oscillation and ω. Evidently, as ω increases, the oscillation of $\gamma_{G,\beta }\left(t\right)$ becomes rapid.

The geometric phase associated with light waves, except for its oscillation, was also studied by other research groups [33,34,35,36]. In particular, Kuratsuji [33] investigated the geometric phase of a polarized light described by the SU(2) coherent state using a different scheme based on the geometry of two interfering beams which were initially split from a source beam. Using a pseudospin concept, he extracted the geometric phase that takes place by interference of two polarization beams having different histories. A nonadiabatic geometric phase of a harmonic oscillator of which parameters vary in time was also reported by Liu et al. [37].

### 2.3. Geometric-Phase Oscillation with One-Photon Processes

The previous analysis of the geometric-phase oscillation can also be extended to general optical phenomena in nano-optics, which are more complicated. We now consider light–matter interactions on a small length scale, which is usually smaller or comparable to the classical limit of light-diffraction. Thanks to the development of nano/mesoscopic engineering techniques, a squeezed state of a light wave within such subwavelength regions can now be obtained and detected [9].

For more detailed understanding of the optical phenomenon along this line from quantum mechanical point of view, let us consider a Hamiltonian $\hat{H}=\hbar \omega \left({\hat{A}}^{†}\hat{A}+1/2\right)$ [22,38] with

(16) $$\hat{A}=\sqrt{\epsilon \omega /\left(2\hbar \right)}\hat{Q}+i\hat{P}/\sqrt{2\epsilon \omega \hbar },$$

where $\hat{Q}=\hat{q}-h_{1}\left(t\right)$ and $\hat{P}=\hat{p}-h_{2}\left(t\right)$ while $h_{i}\left(t\right)$(i=1,2) are some time functions. For simplicity, we consider the time functions of the form

(17) $$h_{1}\left(t\right)=q_{0}\left[1-c_{1}\sin\left(\omega_{1}t\right)\right]\;\;\;\;\;\;\;\;\;h_{2}\left(t\right)=p_{0}\left[1-c_{2}\sin\left(\omega_{2}t\right)\right],$$

where $c_{i}$ are real constants and $\omega_{i}$ are angular frequencies. We assume that $c_{i}\ll 1$ (i=1,2) and $\omega_{i}\ll \omega$ so that we can treat the Hamiltonian as an approximate constant of motion. If we expand this Hamiltonian, it can be represented as

(18) $$\hat{H}=\hat{H}+{\hat{H}}_{p}+H_{0},$$

where $\hat{H}$ is the unperturbed Hamiltonian given in Equation (3), and

(19) $$\begin{array}{ccc} {\hat{H}}_{p} & = & -\left[h_{2}\left(t\right)/\epsilon \right]\hat{p}-\epsilon \omega^{2}h_{1}\left(t\right)\hat{q}, \end{array}$$

(20) $$\begin{array}{ccc} H_{0} & = & h_{2}^{2}/\left(2\epsilon \right)+\epsilon \omega^{2}h_{1}^{2}/2. \end{array}$$

Here, the term ${\hat{H}}_{p}$ represents interaction energies associated with the one-photon mechanisms or linear drivings [22].

By means of the operator $\hat{B}=\mu \hat{A}+\nu {\hat{A}}^{†}$, the squeezed state, which is the eigenstate of the eigenvalue equation $\hat{B}|B\rangle =B|B\rangle$, can be obtained in the configuration space as (see Section 4 which is the Methods section)

(21) $$\langle q|B\rangle ≃\exp\left(ih_{2}\left(t\right)q/\hbar \right)\langle q-h_{1}\left(t\right)|\beta \rangle .$$

The corresponding geometric phase is of the form

(22) $$\gamma_{G,B}\left(t\right)=\int_{0}^{t}\langle B\left(t^{\prime }\right)|i\frac{\partial }{\partial t^{\prime }}|B\left(t^{\prime }\right)\rangle dt^{\prime }+\gamma_{0}.$$

From a procedure similar to the previous case, using Equation (21), we have

(23) $$\begin{array}{cc} \int_{-\infty }^{\infty }dq\left[\langle B|q\rangle \frac{\partial \langle q|B\rangle }{\partial t}\right]= & -i\omega \left(\frac{\left(\mu \alpha -\nu \alpha^{*}\right)\left(\alpha +\alpha^{*}\right)}{\mu -\nu }-\frac{\alpha^{2}}{\left(\mu -\nu \right)\left(\mu^{*}-\nu^{*}\right)}\right)\\ & +\sqrt{\frac{\epsilon \omega }{\hbar }}{\dot{h}}_{1}\left[\frac{1}{\sqrt{2}}\frac{\mu +\nu }{\mu -\nu }\left(\alpha +\alpha^{*}\right)-\sqrt{2}\frac{\mu \alpha +\nu \alpha^{*}}{\mu -\nu }\right]\\ & +\frac{i{\dot{h}}_{2}}{\hbar }\left(h_{1}+\sqrt{\frac{\hbar }{2\epsilon \omega }}\left(\alpha +\alpha^{*}\right)\right). \end{array}$$

By utilizing this, the geometric phase introduced in Equation (22) can be straightforwardly computed to be

(24) $$\begin{array}{ccc} \gamma_{G,B}\left(t\right) & = & \gamma_{G,\beta }\left(t\right)+\gamma_{G,\mathrm{AT}}\left(t\right), \end{array}$$

where the first term is just the one given in Equation (14) and an additional term is given by

(25) $$\gamma_{G,\mathrm{AT}}\left(t\right)=\sqrt{\frac{\epsilon \omega }{\hbar }}\left[\frac{i}{\sqrt{2}}\frac{\mu +\nu }{\mu -\nu }Z_{1}-\frac{\sqrt{2}}{\mu -\nu }Z_{2}\right]-\frac{1}{\hbar }\left(Z_{3}+\sqrt{\frac{\hbar }{2\epsilon \omega }}Z_{4}\right),$$

with

(26) $$\begin{array}{ccc} Z_{1} & = & c_{1}q_{0}\alpha_{0}\omega_{1}\left(\frac{2\omega }{\omega^{2}-\omega_{1}^{2}}\sin\theta -\frac{\sin[\left(\omega -\omega_{1}\right)t+\theta ]}{\omega -\omega_{1}}-\frac{\sin[\left(\omega +\omega_{1}\right)t+\theta ]}{\omega +\omega_{1}}\right), \end{array}$$

(27) $$\begin{array}{cc} Z_{2}= & \frac{c_{1}q_{0}\alpha_{0}\omega_{1}}{\omega^{2}-\omega_{1}^{2}}\{\omega \left[\left(\mu e^{-i(\omega t+\theta )}-\nu e^{i(\omega t+\theta )}\right)\cos\left(\omega_{1}t\right)-\mu e^{-i\theta }+\nu e^{i\theta }\right]\\ & +i\left(\mu e^{-i(\omega t+\theta )}+\nu e^{i(\omega t+\theta )}\right)\omega_{1}\sin\left(\omega_{1}t\right)\}, \end{array}$$

(28) $$\begin{array}{cc} Z_{3}= & \frac{1}{2}c_{1}c_{2}p_{0}q_{0}\omega_{2}\left(\frac{2\omega_{1}}{\omega_{1}^{2}-\omega_{2}^{2}}-\frac{\cos[\left(\omega_{1}-\omega_{2}\right)t]}{\omega_{1}-\omega_{2}}-\frac{\cos[\left(\omega_{1}+\omega_{2}\right)t]}{\omega_{1}+\omega_{2}}\right)\\ & -c_{2}p_{0}q_{0}\sin\left(\omega_{2}t\right), \end{array}$$

(29) $$\begin{array}{ccc} Z_{4} & = & c_{2}p_{0}\alpha_{0}\omega_{2}\left(\frac{2\omega }{\omega^{2}-\omega_{2}^{2}}\sin\theta -\frac{\sin[\left(\omega -\omega_{2}\right)t+\theta ]}{\omega -\omega_{2}}-\frac{\sin[\left(\omega +\omega_{2}\right)t+\theta ]}{\omega +\omega_{2}}\right). \end{array}$$

**Figure 4:**
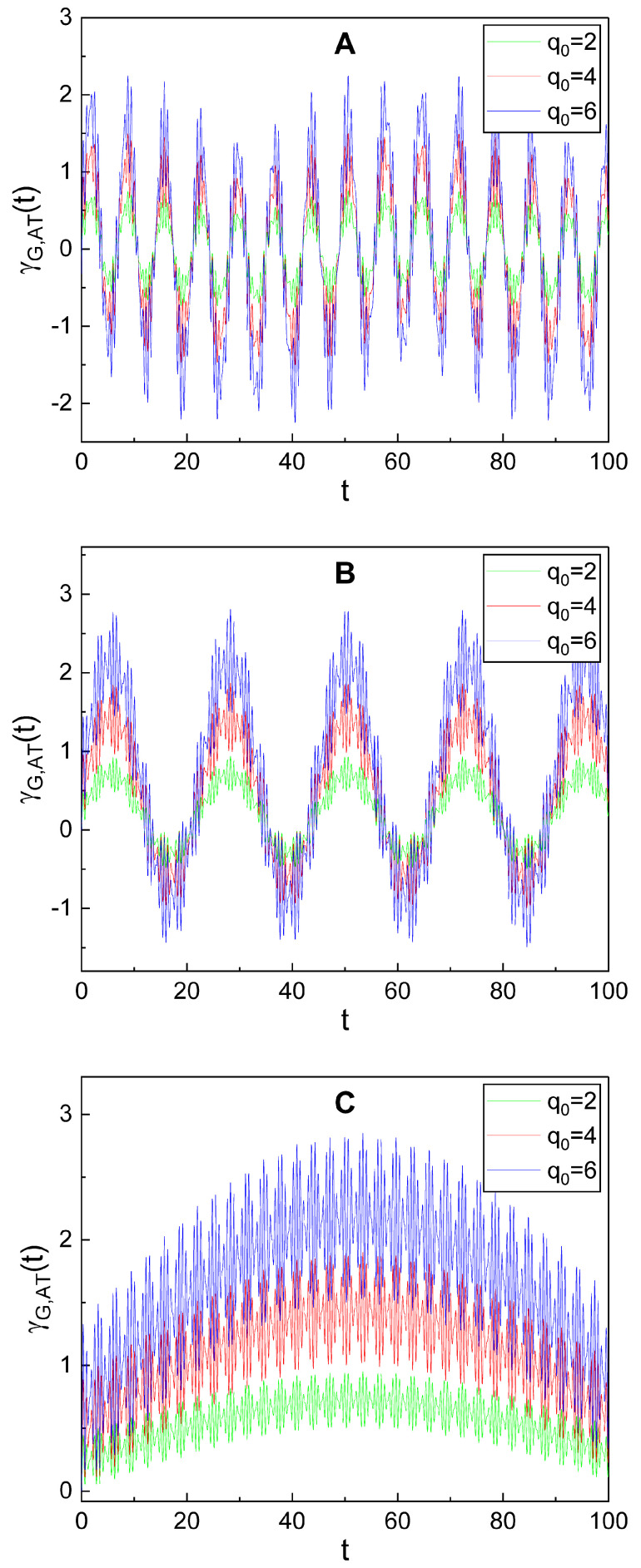
Temporal evolution of the additional term, $\gamma_{G,\mathrm{AT}}\left(t\right)$, of the geometric phase (Equation (25)) for several different values of $q_{0}$. We used $\omega_{2}=0.90$ for (**A**), $\omega_{2}=0.28$ for (**B**), and $\omega_{2}=0.03$ for (**C**). Other values used in the plot are common and they are given by $\omega_{1}=1$, $\xi_{0}=1$, λ=0, ω=10, φ=θ=0, $\gamma_{0}=0$, $\alpha_{0}=5$, $p_{0}=5$, $c_{1}=c_{2}=0.05$, ϵ=1, and ℏ=1. All curves show novel geometric-phase oscillations associated with the one-photon processes.

Detailed time behavior of the additional term $\gamma_{G,\mathrm{AT}}\left(t\right)$ alone has been illustrated in Figure 4. From this, we see that $\gamma_{G,\mathrm{AT}}\left(t\right)$ also oscillates over time, but with a relatively small-scale amplitude. Although the time evolution of $\gamma_{G,\mathrm{AT}}\left(t\right)$ exhibits a somewhat complicated behavior, we can confirm from careful observation of the panels in Figure 4 that its oscillation is periodic. The oscillation of $\gamma_{G,\mathrm{AT}}\left(t\right)$ is composed of a main oscillation whose period is relatively large and a sub-oscillation with a very small period. The amplitudes of both the main and the sub-oscillations increase as $q_{0}$ becomes large. We can also confirm, by comparing the three panels in Figure 4 to each other, that the oscillation pattern of $\gamma_{G,\mathrm{AT}}\left(t\right)$ is very different depending on the value of $\omega_{2}$.

The sub-oscillation exhibits a sort of beating-like oscillatory behavior. A careful inspection reveals that such an outcome is especially prominent in the case of Figure 4C. The analysis for the contributions of the components of Equation (25) to the oscillation of the geometric phase, including its beating behavior, may be interesting. We see from Equation (25) that $\gamma_{G,\mathrm{AT}}\left(t\right)$ is composed of four terms which are relevant to the last four terms of Equation (23) in order. Note that the time behavior of *j*th term(j=1,2,3,4) is determined by the formula of $Z_{j}$. Let us denote *j*th term in Equation (25) as $\gamma_{G,\mathrm{AT}}^{\left(j\right)}\left(t\right)$ for convenience.

**Figure 5:**
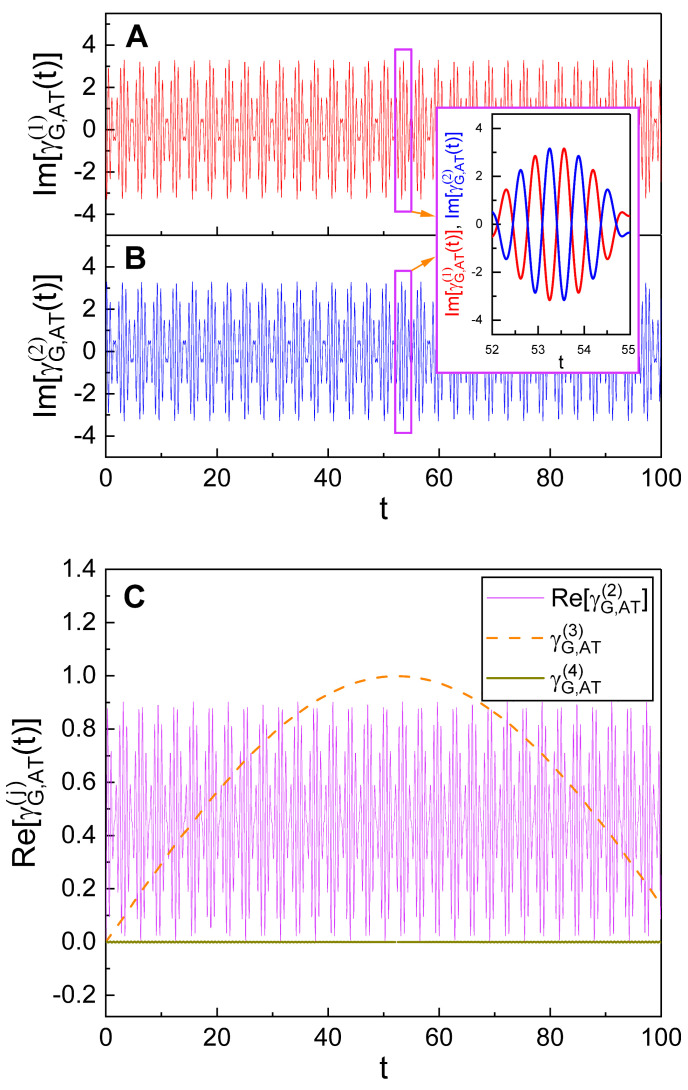
Temporal evolutions of the components $\gamma_{G,\mathrm{AT}}^{\left(j\right)}\left(t\right)$ of the added geometric phase $\gamma_{G,\mathrm{AT}}\left(t\right)$. Imaginary parts Im[γG,AT(1)(t)] and Im[γG,AT(2)(t)] are given in (**A**,**B**), respectively, whereas all real parts are given in (**C**). The inset given between (**A**,**B**) is a graphic of Im[γG,AT(j)(t)] with j=1 (red curve) and j=2 (blue curve), which is enlarged along *t*-axis within 52≤t≤55. We used $q_{0}=4$, while the same values of Figure 4C were used for all other parameters.

For the case of Figure 4, both μ and ν are real numbers because we have chosen λ=φ=0 in that case. Under that choice, the first term $\gamma_{G,\mathrm{AT}}^{\left(1\right)}\left(t\right)$ becomes a purely imaginary number while the second term $\gamma_{G,\mathrm{AT}}^{\left(2\right)}\left(t\right)$ remains as a complex number. On the other hand, the last two terms are independent of μ and ν and are always real. We have depicted the time behavior of the four terms separately for real and imaginary parts in Figure 5 with the choice of the same values of parameters as those of Figure 4C. We see from Figure 5A,B that the imaginary part of $\gamma_{G,\mathrm{AT}}^{\left(2\right)}\left(t\right)$ exactly cancels out $\gamma_{G,\mathrm{AT}}^{\left(1\right)}\left(t\right)$ which is purely imaginary in this case. We can also show this mathematically from an analytical (or numerical) evaluation. Hence, the resulting geometric phase is real, as expected. Figure 5C shows that the real part of $\gamma_{G,\mathrm{AT}}^{\left(2\right)}\left(t\right)$ is responsible for the sub-oscillation of the geometric phase, including its beating behavior, whereas $\gamma_{G,\mathrm{AT}}^{\left(3\right)}\left(t\right)$ is responsible for the main oscillation. On the other hand, the last term $\gamma_{G,\mathrm{AT}}^{\left(4\right)}\left(t\right)$ is negligible.

In this case, the beating behavior of the sub-oscillation is determined by the real part of $Z_{2}$ which governs the time behavior of $\gamma_{G,\mathrm{AT}}^{\left(2\right)}\left(t\right)$. A minor evaluation, under the assumption that μ and ν are real numbers, gives the real part of $Z_{2}$ in a simple form:

(30) Re[Z2]=Z2,0[ω+cos(ω−t+θ)+ω−cos(ω+t+θ)+constant],

where $\omega_{\pm }=\omega \pm \omega_{1}$ and $Z_{2,0}=c_{1}q_{0}\alpha_{0}\omega_{1}\left(\mu -\nu \right)/\left[2\left(\omega^{2}-\omega_{1}^{2}\right)\right]$. Because of the given condition $\omega_{1}\ll \omega$, the difference between $\omega_{+}$ and $\omega_{-}$ is quite small. Thus, we confirm from Equation (30) that the beating behavior of the sub-oscillation originates from the coupling of two sinusoidal oscillations whose frequencies are slightly different from each other.

**Figure 6:**
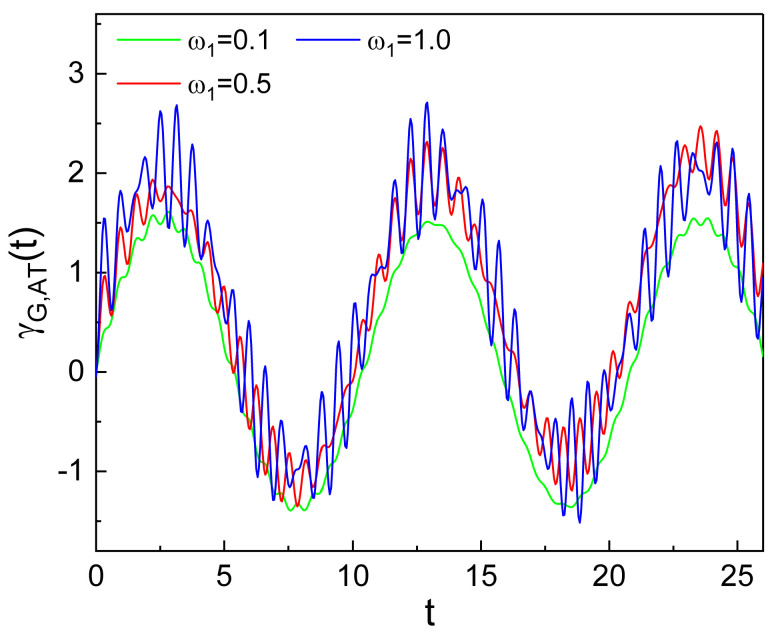
Enlarged view of the temporal evolution of the additional term, $\gamma_{G,\mathrm{AT}}\left(t\right)$, of the geometric phase for several different values of $\omega_{1}$. We used $\omega_{2}=0.6$, $\xi_{0}=1$, λ=0, ω=10, φ=θ=0, $\gamma_{0}=0$, $\alpha_{0}=5$, $q_{0}=6$, $p_{0}=5$, $c_{1}=c_{2}=0.05$, ϵ=1, and ℏ=1. From this figure, we can clearly confirm the main and sub-oscillations in the supplemental geometric-phase oscillation produced by the one-photon processes.

We can see the time behaviors of the main and sub-oscillations more distinctly from Figure 6. It shows the dependence of $\gamma_{G,\mathrm{AT}}\left(t\right)$ on $\omega_{1}$. While the effects of $\omega_{1}$ on the main oscillation are not so significant, the pattern of the sub-oscillation is remarkably different depending on $\omega_{1}$. The amplitude of the sub-oscillation increases as $\omega_{1}$ grows, showing its delicate appearance which is an intricate rapid oscillation. Similar interpretation of the geometric phase evolution is possible when we vary $p_{0}$ and $\omega_{2}$ instead of $q_{0}$ and $\omega_{1}$. Consequently, we conclude that the linear driving induces a small scale additional oscillation of the geometric phase.

## 3. Conclusions

The geometric phase that arises in nano-optics with the squeezed state has been investigated rigorously by using quantum wave mechanics. We have confirmed that such a phase for a light wave oscillates in time with a high amplitude, while the envelope of the oscillation increases linearly as time goes by. The time behavior of the geometric-phase oscillation has been analyzed in detail from various illustrations. As the degree of squeezing for *q*-quadrature increases, the oscillation amplitude grows. Thanks to this property, we can adjust the amplitude of the oscillation by tuning the squeezing parameters. The period of the geometric-phase oscillation is roughly half of that of the probability-density oscillation.

For the case where μ=1 and ν=0, which yields the coherent state, such an oscillation disappears, but the geometric phase still exhibits a linear increase over time. Hence, we conclude that the geometric-phase oscillation originates purely from the effects of the squeezing of the quadrature.

We have extended our development to the squeezed state with one-photon processes in nano-optics. In that case, an additional term appears in the geometric phase, as well as the existing term that corresponds to the unperturbed wave. Such an added term gives a small-scale supplemental geometric-phase oscillation which is composed of the main oscillation and a sub-oscillation. The oscillation period of the main oscillation is relatively large while that of the sub-oscillation is very small. The time behaviors of both the main and the sub-oscillations have been analyzed in detail. In particular, we have confirmed that the sub-oscillation exhibits a beating-like behavior in time.

Geometric-phase oscillations [39,40], as well as the Aharonov–Bohm oscillations [41,42] and the de Haas-van Alphen oscillations [43], are novel physical phenomena which merit great interest in the field of quantum physics and chemistry [44]. As a matter of fact, for a system of a single-molecule magnet that is strongly coupled to metallic leads, one can generate/quench the Kondo resonance by means of the interference that takes place via the geometric-phase oscillation [39]. Indeed, geometric-phase oscillations serve as a new avenue for research in the context of quantum theory associated with nonclassical states.

The oscillation of the geometric phase affects the pattern of the interference [4,18,45]. This is accompanied by many interesting physical phenomena, such as conductance fluctuations [46], antilocalizations [47], and nondissipative current flows [48]. The study of the interference in phase space was started by Wheeler in the context of squeezed states [49]. In fact, such interference in squeezed states is responsible for another novel phenomenon which is oscillations in photon number distribution (PND). Schleich [28,50] and Dutta [51] with their collaborators explained the oscillation of PND, emphasizing it as a signature of nonclassicality of the state. Perhaps the geometric-phase oscillation in addition to the PND oscillation is a key feature which should be considered in quantum-state engineering with the squeezing for both bare and superposition states [4,18,45].

## 4. Methods

### 4.1. Wave function and Expectation Values

By solving the eigenvalue equation $\hat{b}|\beta \rangle =\beta |\beta \rangle$ in a straightforward way in the configuration space, we have

(31) 〈q|β〉=Nexp[−Ξ(q)],

where

(32) $$\begin{array}{ccc} N & = & {\left[\frac{\epsilon \omega }{\hbar \pi \left(\mu -\nu \right)\left(\mu^{*}-\nu^{*}\right)}\right]}^{1/4}, \end{array}$$

(33) $$\begin{array}{ccc} \Xi (q) & = & \frac{1}{\hbar }\left(\frac{\mu +\nu }{\mu -\nu }\frac{\epsilon \omega }{2}q^{2}-\sqrt{2\hbar \epsilon \omega }\frac{\mu \alpha +\nu \alpha^{*}}{\mu -\nu }q\right)+\frac{{\left|\alpha \right|}^{2}+\alpha^{2}}{2\left(\mu -\nu \right)\left(\mu^{*}-\nu^{*}\right)}. \end{array}$$

This is the wave function in the squeezed state, which is necessary for evaluating the corresponding geometric phase. This is the particular case of the squeezed state reported in [52] for a light wave in a more general medium in which the electric conductivity cannot be ignored. A similar type of the squeezed state is also given in [53]. Notice that, for the case of μ→1 and ν→0, Equation (31) reduces to the coherent state represented in Equation (2.5.38) of [54].

We can see from Equation (1) that *q* is a time function which is related to the amplitude of the wave at a given time; for this reason, 〈q|β〉 in Equation (31) is irrelevant to the position of the wave. In general, field quantization for a single-mode plane wave is carried out using the Hamiltonian represented in terms of only time function *q* and its canonical conjugate variable *p* (cf. Equation (2.10) of [29] and Equation (6) of [52]).

The expectation values of operators necessary in the development of the theory in the squeezed state are given by [55]

(34) $$\begin{array}{c} \langle \beta |\hat{a}|\beta \rangle =\alpha , \end{array}$$

(35) $$\begin{array}{c} \langle \beta |{\hat{a}}^{2}|\beta \rangle =\alpha^{2}-\mu \nu , \end{array}$$

(36) $$\begin{array}{c} \langle \beta |{\hat{a}}^{†}\hat{a}|\beta \rangle ={\left|\alpha \right|}^{2}+{\left|\nu \right|}^{2}. \end{array}$$

In addition, the expectation values $\langle \beta |{\hat{a}}^{†}|\beta \rangle$ and $\langle \beta |{\left({\hat{a}}^{†}\right)}^{2}|\beta \rangle$ are complex conjugates of the results of Equations (34) and (35), respectively. Using these relations we can easily confirm that the fluctuations of canonical variables defined as ${\left(\Delta y\right)}_{\beta }=\left[\langle \beta \right|{\hat{y}}^{2}|\beta \rangle -\left(\langle \beta \right|\hat{y}{\left|\beta \rangle \right)}^{2}{]}^{1/2}$ where y=q,p are given by Equation (6) in the text.

### 4.2. Geometric Phase Obtained from p-Representation

The *p*-space eigenstate can be obtained from the Fourier transformation of the form

(37) $$\langle p|\beta \rangle =\frac{1}{\sqrt{2\pi \hbar }}\int_{-\infty }^{\infty }\langle q|\beta \rangle e^{-ipq/\hbar }dq.$$

From a minor evaluation after inserting Equation (31) in the above equation, we have

(38) $$\langle p|\beta \rangle =\tilde{N}\exp\left[-\tilde{\Xi }\left(p\right)\right],$$

where

(39) $$\begin{array}{ccc} \tilde{N} & = & {\left[\frac{\mu -\nu }{\epsilon \omega (\mu +\nu )}\right]}^{1/2}N, \end{array}$$

(40) $$\begin{array}{cc} \tilde{\Xi }\left(p\right)= & \frac{1}{\hbar }\left(\frac{\mu -\nu }{\mu +\nu }\frac{p^{2}}{2\epsilon \omega }+i\sqrt{\frac{2\hbar }{\epsilon \omega }}\frac{\mu \alpha +\nu \alpha^{*}}{\mu +\nu }p\right)+\frac{{\left|\alpha \right|}^{2}+\alpha^{2}}{2\left(\mu -\nu \right)\left(\mu^{*}-\nu^{*}\right)}\\ & -\frac{{\left(\mu \alpha +\nu \alpha^{*}\right)}^{2}}{\mu^{2}-\nu^{2}}. \end{array}$$

Now we apply another identity operator, $\int_{-\infty }^{\infty }dp|p\rangle \langle p|=I$, into Equation (10) as

(41) $$\gamma_{G,\beta }\left(t\right)=i\int_{0}^{t}dt^{\prime }\int_{-\infty }^{\infty }dp\langle \beta \left(t^{\prime }\right)|p\rangle \frac{\partial }{\partial t^{\prime }}\langle p|\beta \left(t^{\prime }\right)\rangle +\gamma_{0}.$$

The integration with respect to *p*, using Equation (38) with Equations (39) and (40), gives

(42) $$\begin{array}{cc} \int_{-\infty }^{\infty }dp\left[\langle \beta |p\rangle \frac{\partial \langle p|\beta \rangle }{\partial t}\right]= & -i\omega (\frac{\left(\mu \alpha -\nu \alpha^{*}\right)\left(\alpha^{*}-\alpha \right)}{\mu +\nu }+\frac{2(\mu^{2}\alpha^{2}-\nu^{2}\alpha^{*2})}{\mu^{2}-\nu^{2}}\\ & -\frac{\alpha^{2}}{\left(\mu -\nu \right)\left(\mu^{*}-\nu^{*}\right)})\\ & =-i\omega \left(\frac{\left(\mu \alpha -\nu \alpha^{*}\right)\left(\alpha +\alpha^{*}\right)}{\mu -\nu }-\frac{\alpha^{2}}{\left(\mu -\nu \right)\left(\mu^{*}-\nu^{*}\right)}\right). \end{array}$$

The result, Equation (42), is exactly the same as that which was derived in *q*-representation (see Equation (12)). The remaining integration related to *t* is just the same repeating of that in the *q*-representation. Hence, we can conclude that the geometric phase is space-independent; i.e., the geometric phase derived in *p* space is identical to that derived in *q* space.

### 4.3. Method for Deriving Squeezed State for the Hamiltonian $\hat{H}$

From Hamiltonian dynamics with $\hat{H}$ that belongs to the one-photon process, we have the corresponding classical equations of motion as

(43) $$\begin{array}{ccc} \ddot{q}+\omega^{2}q & = & \omega^{2}q_{0}\left[1-f_{1}\left(t\right)\right], \end{array}$$

(44) $$\begin{array}{ccc} \ddot{p}+\omega^{2}p & = & \omega^{2}p_{0}\left[1-f_{2}\left(t\right)\right], \end{array}$$

where

(45) $$\begin{array}{ccc} f_{1}\left(t\right) & = & c_{1}\sin\left(\omega_{1}t\right)-\frac{c_{2}p_{0}\omega_{2}}{\epsilon q_{0}\omega^{2}}\cos\left(\omega_{2}t\right), \end{array}$$

(46) $$\begin{array}{ccc} f_{2}\left(t\right) & = & c_{2}\sin\left(\omega_{2}t\right)+\frac{c_{1}\epsilon q_{0}\omega_{1}}{p_{0}}\cos\left(\omega_{1}t\right). \end{array}$$

If we denote the solution of Equation (43) (Equation (44)) as $Q_{\mathrm{cl}}$ ($P_{\mathrm{cl}}$), it consists of the complementary function $Q_{c}$ ($P_{c}$) and the particular solution $Q_{p}$ ($P_{p}$) [56]:

(47) $$\begin{array}{ccc} Q_{\mathrm{cl}}\left(t\right) & = & Q_{c}\left(t\right)+Q_{p}\left(t\right), \end{array}$$

(48) $$\begin{array}{ccc} P_{\mathrm{cl}}\left(t\right) & = & P_{c}\left(t\right)+P_{p}\left(t\right). \end{array}$$

In this case, each term of complementary functions is given by

(49) $$\begin{array}{ccc} Q_{c} & = & C\cos(\omega t+\theta ), \end{array}$$

(50) $$\begin{array}{ccc} P_{c} & = & -\epsilon \omega C\sin(\omega t+\theta ). \end{array}$$

Here, $Q_{c}$ is actually the same as $q_{\mathrm{cl}}$ which we have previously introduced as the classical solution for the unperturbed light wave (see Equation (2)).

The particular solutions of Equations (43) and (44) are given by

(51) $$\begin{array}{ccc} Q_{p} & = & q_{0}-\frac{\omega^{2}q_{0}c_{1}}{\omega^{2}-\omega_{1}^{2}}\sin\left(\omega_{1}t\right)+\frac{c_{2}p_{0}\omega_{2}}{\epsilon (\omega^{2}-\omega_{2}^{2})}\cos\left(\omega_{2}t\right), \end{array}$$

(52) $$\begin{array}{ccc} P_{p} & = & p_{0}-\frac{\omega^{2}p_{0}c_{2}}{\omega^{2}-\omega_{2}^{2}}\sin\left(\omega_{2}t\right)-\frac{\omega^{2}c_{1}\epsilon q_{0}\omega_{1}}{\omega^{2}-\omega_{1}^{2}}\cos\left(\omega_{1}t\right). \end{array}$$

If we consider the assumptions $c_{i}\ll 1$ (i=1,2) and $\omega_{i}\ll \omega$ Equations (51) and (52) to be

(53) $$\begin{array}{ccc} Q_{p}≃h_{1}\left(t\right) & = & q_{0}\left[1-c_{1}\sin\left(\omega_{1}t\right)\right], \end{array}$$

(54) $$\begin{array}{ccc} P_{p}≃h_{2}\left(t\right) & = & p_{0}\left[1-c_{2}\sin\left(\omega_{2}t\right)\right]. \end{array}$$

The eigenvalue equation for $\hat{A}$ can be expressed as $\hat{A}|A\rangle =A|A\rangle$ where *A* is the eigenvalue and |A〉 is the eigenstate. From Equation (16), we can express the eigenvalue in the form

(55) $$A=\sqrt{\epsilon \omega /\left(2\hbar \right)}Q+iP/\sqrt{2\epsilon \omega \hbar }.$$

Note that *Q* and *P* can be written as

(56) $$\begin{array}{ccc} Q & = & q-h_{1}≃Q_{c}, \end{array}$$

(57) $$\begin{array}{ccc} P & = & p-h_{2}≃P_{c}. \end{array}$$

In the derivation of the above consequences, *q* and *p* have been replaced with $Q_{\mathrm{cl}}$ and $P_{\mathrm{cl}}$ respectively, without loss of generality, and the relations in Equations (47), (48), (53) and (54) have been used. Thus, Equation (55) can be reexpressed in terms of $Q_{c}$ and $P_{c}$ such that

(58) $$A=\sqrt{\epsilon \omega /\left(2\hbar \right)}Q_{c}\left(t\right)+iP_{c}\left(t\right)/\sqrt{2\epsilon \omega \hbar }.$$

Using Equations (49) and (50), it becomes

(59) $$A=\sqrt{\frac{\epsilon \omega }{2\hbar }}Ce^{-i(\omega t+\theta )}.$$

From the eigenvalue equation

(60) $$\hat{B}|B\rangle =B|B\rangle ,$$

we directly have

(61) $$\begin{array}{cc} \langle q|B\rangle = & \sqrt[{4}]{\frac{\epsilon \omega }{\hbar \pi \left(\mu -\nu \right)\left(\mu^{*}-\nu^{*}\right)}}\exp[-\frac{1}{\hbar }(\frac{\mu +\nu }{\mu -\nu }\frac{\epsilon \omega }{2}{\left(q-h_{1}\right)}^{2}\\ & -\sqrt{2\hbar \epsilon \omega }\frac{\mu A+\nu A^{*}}{\mu -\nu }\left(q-h_{1}\right)-ih_{2}q)-\frac{{\left|A\right|}^{2}+A^{2}}{2\left(\mu -\nu \right)\left(\mu^{*}-\nu^{*}\right)}]. \end{array}$$

By the way, if we compare the above equation with Equation (31) under the consideration that the formula of *A* in Equation (59) is the same as that of α in Equation (4), we confirm that 〈q|B〉 can be represented as Equation (21) in the text.

## Figures and Tables

**Table 1:** Fluctuations of the canonical variables *q* and *p* for each curve in panels A and B in Figure 2. These are evaluated from Equation (6) with the option of ϵ=1 and ℏ=1.

	Figure 2A			Figure 2B		
**No.**	$\xi_{0}$	${\left(\Delta q\right)}_{\beta }$	${\left(\Delta p\right)}_{\beta }$	φ	${\left(\Delta q\right)}_{\beta }$	${\left(\Delta p\right)}_{\beta }$
1	0.4	0.47399	1.05488	0.01π	0.12606	5.22421
2	0.6	0.38807	1.28843	0.02π	0.18996	5.22227
3	0.8	0.31772	1.57370	0.03π	0.26404	5.21905
4	1.0	0.26013	1.92212	0.04π	0.34169	5.21455
5	1.2	0.21298	2.34768	0.05π	0.42089	5.20875
6	1.4	0.17437	2.86746	0.06π	0.50085	5.20167
7	1.6	0.14276	3.50232	0.07π	0.58118	5.19331
